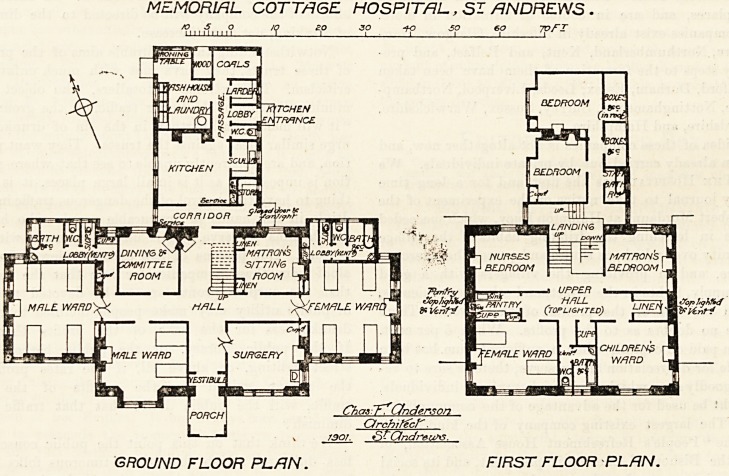# Cottage Hospital, St. Andrews

**Published:** 1901-08-10

**Authors:** 


### THE INSTITUTIONAL WORKSHOP.

### COTTAGE HOSPITAL, ST. ANDREWS.

THE existing hospital was opened in 1865, in remembrance of Lady William Douglas, and it was added to fifteen years later. This building has been sold, and a new hospital is about to be erected at the east end of the Abbey Park. The site chosen is well adapted for its purpose, as it is sheltered from the north and east winds, and it will give a south-south-west exposure to the new wards. The ground slopes towards the south, and the sub-soil is dry sand.

The main part of the hospital is two stories in height, and it is formed of a centre and two wings. The former, on its ground-floor, contains committee-room, matron's sitting-room, surgery, which will be used as an operation-room, and a male ward for two beds. As this ward is incorporated with the centre, it is impossible to provide it with proper cross-ventilation, but it has a window in its west side as well as a large window to the south, and by these means a current of air through part of the ward is possible. The male ward for four beds constitutes the west wing, and the female ward for the same number of beds is the east wing. Both these wards would have been well arranged had it not been for the position of the sanitary block, which is placed alongside the ward, occupying the whole of one side except that part taken up by a small window. Evidently the sanitary blocks ought to have been placed at right angles to their present positions, and cut off from the ward by efficiently cross-ventilated passages; while the kitchen block should have been ran further north by turning its ventilated passages from east and west to north and south. The extra cost of this arrangement would have been infinitesimal, and it would have immensely improved both the wards and the sanitary blocks. The hall is of fair size for a small hospital, and, being fitted up with a fireplace, it can be used as extra day space by the patients during wet weather. The kitchen and its offices, with laundry, are well arranged except the w.c., which is too closely incorporated with the block.

The first floor contains matron's bedroom, nurses' bedroom, servants' rooms, and servants' bath-room; patients' bath-room and closet, the latter ventilated to roof, linen room and pantry. On this floor are also a female ward for two beds and a children's ward. These wards occupy respectively the south-west and south-east corners of the centre; consequently the same remarks, as regards window ventilation, already made when describing the two-bedded ward on ground floor, apply to them also.

In the matter of construction everything seems to have been arranged on a liberal scale. The main walls are of local freestone and are two feet thick, and all the walls are strapped, lathed and plastered inside with three-coat work. Roofs are covered with slate. All angles are rounded and special concave skirtings are provided in the wards. The floor and walls of the surgery and operation room are covered with tiles; and this room, and all the wards are provided with air inlets and extractors; and the fireplaces are so constructed that they will supply fresh warm-air to the wards. The windows very properly run up to the ceilings, the upper parts being hinged to open inwards, and the lower parts made to slide up or down, with special provision for ventilation at the meeting rails.

The sanitary fittings are of the best description and newest design; and the drainage well carried out. The cost will be under £2,500; and the plan is such that it could be easily and cheaply added to.

The mortuary and ambulance house are entirely separated from the main building, and have an entrance on the high road.

The architect is Mr. C. F. Anderson, of St. Andrews.

MEMORIAL COTTAGE HOSPITAL, ST ANDREWS.

GROUND FLOOR PLAN. FIRST FLOOR PLAN.

## Figures and Tables

**Figure f1:**